# Understanding sexual behaviors of youth from the lens of caregivers, teachers, local leaders and youth in Homabay County, Kenya

**DOI:** 10.1186/s12978-023-01680-2

**Published:** 2023-09-19

**Authors:** Eunice Omanga, Irene Inwani, Kawango Agot, Jasmine Buttolph, Ruth Nduati, Paul Macharia, Jacob Onyango, Ann Kurth

**Affiliations:** 1 Great Lakes University of Kisumu, Kisumu, Kenya; 2 Impact Research and Development Organization, Kisumu, Kenya; 3 University of Nairobi, Nairobi, Kenya; 4 Kenyatta National Hospital, Nairobi, Kenya; 5 Yale University, New Haven, CT USA; 6 United States Agency for International Development (USAID), Washington, DC USA; 7 The New York Academy of Medicin (NYAM), New York, USA

**Keywords:** HIV, Adolescents, Sexual-debut, Transactional sex, Multiple sexual partners

## Abstract

In Kenya similar to other countries in Eastern and Southern Africa There is a disproportionately high burden of the global HIV incidence among youth ages 15–24 years, and where adolescent girls and young women account for up to a third of all incident HIV infections and more than double the burden of HIV compared to their male peers. Previous work has shown early sexual debut as entry point into risks to sexual and reproductive health among young people including STI/HIV acquisition. This was a formative assessment of the local context of three sexual risk behaviors among youth ages of 15–24 years: early sexual debut, multiple sexual partnerships, and age-mixing /intergenerational sex for purposes of informing comprehensive combination HIV intervention program design. We conducted a cross-sectional formative qualitative study in four sub-counties within Homabay county a high HIV prevalence region of Kenya. Participants were recruited through youth groups, schools, government offices and, community gatekeepers using approved fliers, referred to a designated venue for focus group discussion (FGD). After oral informed consent, twelve FGDs of 8–10 participants were carried out. Transcripts and field notes were uploaded to Atlas.ti qualitative data analysis and research software (version 8.0, 2017, ATLAS.ti GmbH). Open coding followed by grouping, categorization of code groups, and thematic abstraction was used to draw meaning for the data. A total of 111 youth participated in the FGD, 65 males and 46 females. The main findings were that youth engaged in early sex for fear of being labeled ‘odd’ by their peers, belief (among both male and female) that ‘practice makes perfect’, curiosity about sex, media influence, need to prove if one can father a child (among male), the notion that sex equals love with some of the youth using this excuse to coerce their partners into premature sex, and the belief that sex is a human right and parents/guardians should not intervene. Male youth experienced more peer-pressure to have sex earlier. Female youths cited many reasons to delay coitarche that included fear of pregnancy, burden of taking care of a baby, and religious doctrines. Having multiple sexual partners and intergenerational sexual relationships were common among the youth driven by perceived financial gain and increased sexual prowess. HIV prevention strategies need to address gender vulnerabilities, as well as promoting a protective environment, hence application of combination prevention methods is a viable solution to the HIV pandemic.

***Trial registration number:*** The study was approved by the KNH/UoN Ethics review committee (KNH/UoN ERC-P73/03/2011) and New York University (NYU Reg no.–00000310).

## Introduction

Despite a global decline in HIV prevalence among youth ages 15–24 years, this group still bears a disproportionately high burden of the global HIV incidence, accounting for 29% of all new infections in 2019 [52]. This underscores the vulnerability of the youth and the need to design strategic prevention programs that are youth-focused. However, adolescence is a transitional period from childhood to adulthood marked by physical, psychological and social changes (United Nations International Children's Emergency Fund [51, 56]. It is characterized by curiosity, risk-taking and experimentation [11, 42] which increase young peoples’ vulnerability to contracting HIV and other sexually transmitted infections (STIs) as well as being vulnerable to health risks associated with premature/unplanned pregnancies and substance abuse [8].

Juma et al. [23] identified factors that increase risky adolescent sexual behaviors as poverty (lack of basic needs, poor housing), school dropouts, transactional sex and early marriages. The study identified poverty as a hindrance to adolescent orphans’ and non-orphans’ access to basic needs. Globally, women continue to carry the greater burden of HIV and in Sub-Saharan Africa this burden is higher among young women [9, 53].

The World AIDS Report [53] reveals that in Eastern and Southern Africa region (ESAR), adolescent girls and young women account for 26% of new infections, and has more than double the risk and burden of HIV compared to their male peers. Similarly, the ESAR has among the highest adolescent fertility rates in the world and with at least 15% of young women aged 15–19 years having started reproduction [45], quantified the differences in HIV/AIDS prevalence between women and men using demographic and health (DHS) data found that 84% (P < 0.001) and 92% (P < 0.001) of the higher prevalence of HIV/AIDS among women in Uganda and Ghana, respectively, was explained by the different distributions of HIV/AIDS risk factors, particularly age at first sex between women and men. A similar study looking at sex differences in HIV prevalence from 18 countries in Sub-Saharan Africa found that female: male HIV prevalence ratio was above one in all countries in at least one survey round for both ages 15–24 years and 25–49 years [20]. The researchers used data from two rounds of Demographic and Health Surveys. The study further revealed that in over 70% of the countries (13 out of 18), the prevalence ratio was higher for the younger age group compared to the age group 25–49 years (3 significant) and this difference in prevalence ratios between the age groups did not change over time.

Similarly, adolescents and young people in Kenya significantly contribute to high HIV burden in the country with young women of ages 15–24 accounting for a third of all new HIV adult infections [19, 34]. It is estimated that 2.6% of female youth aged 15–24 years are living with HIV/AIDS, compared to 1.3% of their male counterparts [26]. HIV prevalence in girls and boys aged 15–19 was 1.2% and 0.5% respectively, while there was a three-fold difference for girls in the 20–24 years’ age group (3.4% females compared to 0.6% males). Progressively, more young men and women are having sex with increase in age, and among those aged 15–19 years, 17.1% females compared to 24.8% males had sexual intercourse in the 12 months preceding the survey; however, in the 20–24 age bracket, 47.4% young women and 66.3% young men had sexual intercourse in the 12 months preceding the survey [26].

Early sexual debut, defined as having one’s first incident of sexual under the age of 15 years [41, 44) and is associated with risks to sexual and reproductive health among young people [3, 39]. It is one of the risk factors for STI/HIV acquisition; youth with early coitarche are more likely to engage in risky sexual behaviors, such as unprotected sex and multiple sexual partners who are also high-risk [43]. Studies have consistently shown compelling evidence for the association between early sexual debut and the risk for HIV infection in sub-Saharan Africa [47–49, 54]. From KDHS [26] data, young men (15–24 years) are almost twice as likely to engage in sexual intercourse before age 15 as compared to young women of the same age (21% against 12%). Additionally, both biological susceptibility and gendered power dynamics disadvantage women and increase their vulnerability to HIV while teen girls commonly have large areas of ectopy [21, 35]. By age 18, more than half (55%) of men and close to half (47%) of women will have had sexual intercourse. Additionally, HIV-related knowledge is higher among young men (66% against 57% for young women), which increases with age. However, this knowledge is not always translated into action by youth and is thus a source of concern as early sexual debut increases the risk of premature pregnancies and STIs (including HIV) by prolonging the prospective period of sexual activity for the individual [48]. While peer pressure is a risk factor for early sexual debut, the main facilitator for delayed coitarche is having supportive relationships with trusted role models, specifically parents, teachers, and spiritual leaders [5]. Empowering youth to decline sex and delay sexual debut by giving them the tools do negotiate these situations is key in addition to keeping them in school—supporting the youth with school fees, sanitary towels, pocket money (Cash transfers).

Multiple partnerships have been associated with increased risk of STI and HIV [4, 13, 37]. Kenyan men in general are almost nine times more likely than women to have multiple sexual partners [26]. Among male adolescents aged 15–24 years, 9.6% reported having had multiple partners (more than 2) in the 12 months preceding the survey, out of which 69. 8% reported using condoms during their last sexual encounter. On the other hand, 1.5% of females aged 15–24 reported having more than two partners in the preceding 12 months out of which only 37.5% reported using condoms during their last sexual encounter. These figures confirm the urgent need to address risky sexual behavior in Kenyan youth. Away from Kenya, a study exploring the association between early sexual debut and sexual partners among Vietnamese women found that those who had sexual debut at age 19 or younger were more than five times likely to have multiple sexual partners compared to women who engaged in sex for the first time after marriage [47].

Researchers have demonstrated that intergenerational sex is common in sub-Saharan Africa, with relationships between older men and young women contributing to the spread of HIV [15, 32, 57]. Due to the high level of poverty in some African countries including Kenya, some young women engage in transactional sex with older men in exchange for personal needs such as sanitary towels, beauty products and even education-related expenses like books and school uniforms. Thus, young girls do not necessarily engage in sexual activities voluntarily but do so due to pressing needs and limited negotiation power and even more limited negotiation skills. Such female youth are therefore more susceptible to HIV infection.

Biomedical, structural and behavioral interventions offer protection against HIV infection [22]. There is growing evidence that strategies that combine these partially protective interventions at different levels (individual, family, community, etc.) may provide enhanced protective effect against HIV [2]. The Project Accept intervention was conducted in one Asian and three African countries where incorporating community based mobilization, voluntary counseling and testing, and post-test counseling returned a four-fold increase in testing and 95% adherence to intervention components [27].

This paper is a formative assessment of three sexual risk behaviors among youth ages of 15–24 years: early sexual debut, multiple sexual partnerships, and age-mixing /intergenerational sex. The aim was to understand local contexts of these practices for purposes of informing comprehensive combination HIV intervention program design.

## Materials and methods

### Study setting

This was a cross-sectional qualitative study carried out in February and March 2011 in four districts in Homa Bay County, a rural neighborhood on the shores of Lake Victoria that is largely inhabited by agricultural and fishing communities. The region had the highest HIV prevalence at the onset of the study, at 13.9% compared to 6.3% nationally [25], with Homabay still having the highest prevalence in 2018, at 19.6% relative to the national prevalence of 4.9% [36].

### Recruitment of study participants

The researchers worked in partnership with the local community through well-established NGO (IMPACT-RDO) that was at that time providing combination HIV prevention to the youth.

A youth Community Advisory Board (CAB) was assembled to facilitate continuous community engagement, comprised of area youth, leaders, teachers, and health providers throughout the District services.

Participants were recruited through youth groups, schools, government offices, community leaders such as chiefs, churches, women’s groups and other community-based organizations.

Those eligible and interested were invited to participate in the focus group discussion both verbally as well as providing them with fliers that indicate the location, date, and time for focus group discussion (FGD).

Each FGD was assigned between 8 to 10 participants and were grouped as follows: male youth 15–17 years, male youth 18–24 years, female youth 15–17 years, female youth 18–24 years, combined male and female youth 15–17 years, combined male and female youth 18–24 years, parents and teachers of youth 15–17 years, parents and teachers of youth 18–24 years, community leaders, district-level government officials, and religious leaders.

Consenting was done orally by the RAs for parents of the younger youth (15–17 years) during recruitment and at the beginning of each FGD for all adult participants. Participants and parents of younger youth were given the consent forms to read for themselves while those who were unable to read had the consent forms read to them. Consent forms were available in English, Kiswahili and the Dholuo (local dialect). The consent forms for parents who could not read and/or write were read for them in the presence of an independent witness. The witness was a literate person chosen by the participant and was not affiliated with the study in any way. Call backs were made in cases where parents were not available during the initial visits.

### Data collection

A structured FGD guide was used to explore youth sexual behavior, HIV prevention (including preferred location and delivery of prevention packages), girl child education and youth substance abuse. The guide was translated from English into Kiswahili and Dholuo and back-translated into English. One moderator led all the discussions while two research assistants took detailed notes. A language understood by all participants was used; however, in most cases, all the 3 languages were used interchangeably when clarifying the questions or when participants were responding.

### Ethical approval

Study procedures were approved by the Kenyatta National Hospital/ Nairobi University Ethics Review Committee and New York University Institutional Review Board. All participants provided oral consent to participate in the study after either reading or being read to the informed consent document. Consent to publish de-identified data was obtained from the respondents.

### Data management and analysis

All discussions were digitally recorded and transcribed verbatim, supported with detailed field notes to aid in understanding the recorded data. Transcription/translation was done concurrently by two of the RAs, one of whom was the FGD moderator. Transcripts and field notes were uploaded to Atlas.ti qualitative data analysis and research software (version 8.0, 2017, ATLAS.ti GmbH), for analysis. Coding was done inductively to draw meaning from the data through open coding followed by code grouping, categorization of code groups, and thematic abstraction. Initial coding was done utilizing broad descriptive codes generated by review of a random sample of three transcripts and FGD guides. Theoretical memos created during this process were used to refine codes and discussed with the PI and other investigators before returning to the raw transcripts to recode using the new schema. The resulting subset of data was organized to visualize the relevance of codes across the different groups of respondents. Findings are presented by theme, illustrated by respondent quotes.

Focus groups sessions were audio-taped and stored using study IDs only (no names or identifier were elicited during the FGDs). Recordings were referred to as needed in summarizing the interview data, after which they were destroyed within one year following the study write-up and dissemination.

### Study limitations

A limitation of this study is that the findings may not be generalizable to the general population in Kenya because it covers only one of the 47 counties. However, the study offers an account of youth sexual behaviors and identifies facilitators and barriers to behavior change as perceived by the youth, their parents and teachers and other stake holders in Homabay County that can be applicable to similar communities.

## Results

Twelve FGDs were conducted, with each session comprising of 8 -10 participants. Table 1 presents a distribution of the FGD participants.

**Table 1:** Data Collection Events

Category	Composition		Total
	Males	Females	
Male youth 15–17 years	10	–	10
Male youth 18–24 years	10	–	10
Female youth 15–17 years	–	9	9
Female youth 18–24 years	–	8	8
Combined male & female youth 15–17 years	5	5	10
Combined male & female youth 18–24 years	5	5	10
Male and female students combined 17–24 years	5	3	8
Parents & Teachers of youth 15–17 years	3	7	10
Parents & Teachers of youth 18–24 years	4	4	8
Community Leaders	9	1	10
District officials	7	2	9
Religious leaders	7	2	9
Total	65	46	111

Findings are organized according to two main themes that emerged from data analysis: whether the three risky sexual behaviors (early sexual debut, having multiple sexual partners and engaging in intergenerational sex) are exhibited by the youth in the study community, and reasons for and against the sexual behaviors. Different categories emerged for each theme which are summarized and supported by appropriate quotations.

### Early sexual debut/delaying coitarche

The perceived average age of sexual debut was from 12 to 16 years, with peer pressure cited as the main obstacle to delaying sexual debut especially for male youth. Majority of the youth engaged in early sex for fear of being labeled ‘odd’ when their friends/colleagues bragged about their sexual experiences. Other reasons identified for early sexual debut in youth included: the belief (among both male and female) that ‘practice makes perfect’, curiosity about sex, media influence, need to prove if one can father a child (among male), the notion that sex equals love with some of the youth using this excuse to coerce their partners into premature sex, and the belief that sex is a human right and parents/guardians should not intervene. Peer pressure was most mentioned as a facilitator of early sexual debut as by some of the participants cited below.

“*For example when someone is telling me to wait but in the environment I come from I see my age mates doing it, I feel am the odd one out*” Combined male and female youths 15-17 years.

“*I think also peer pressure. When people are at a group somebody can come with an idea like she has a boyfriend who can provide for whatever she wants, then other girls can also be lured to start those activities*” Combined male and female youths 15 - 17 years.

“*Most of young people think if they wait till that required age, most males say they will not have experience about sex. So he has to start earlier such that when he reaches marriage age he has some experience*” Female youth 18-24 years.

“*I think the youth can see that some people might think that they are abnormal. You know like in this society when you have a boy who is not associating with girls or his peer you will start getting worried*” Parents and teachers of youth 18 -24 years.

It was interesting to note that adults (parents and teachers) reported that 90–100% of youth aged 15–24 were sexually active while the youth estimated that 50–70% of them were sexually active.

### Gender differences in delayed sexual debut

Male youth felt more pressure to have sex earlier compared to females who had more reasons to delay coitarche. Participants identified reasons for delayed sexual debut among female youth including: avoiding pregnancy, wanting to complete school, observance of religious doctrine, waiting to secure employment, knowledge of the consequences of premature sex (HIV/AIDS, pregnancy) and pride of keeping their virginity until marriage. For the male youth, prevention of STIs especially HIV was the most cited reason for delaying coitarche. On the other hand, even though young women experience pressure from their male sexual partners to engage in sex as a proof of their love for them, fear of pregnancy and the burden of taking care of a baby are reasons for delayed sexual debut among young women as elaborated by one of the participants below:

“*I can say that women have it in mind that having sex at an early stage they are at a high risk but the pressure from men; you have a boyfriend that can tell you that ‘you don’t love me and that is why you don’t want to have sex with me,’ so the pressure that she proves to the man that she loves him. So things like that but they do fear because they fear that they will give birth and there is nobody who will take care of the baby so they will only regret once they have done it*”. Females youth 18-24 years.

There were many misconceptions fueled by myths for and against early sexual debut for women The participants shared that there are those who think that when a woman does not have sex early her birth canal will be blocked or be too narrow and she will have problems delivering a baby when the time comes. Another belief is that a woman who starts having sexual intercourse at a young age will not be able to have children.

*“A disadvantage is during child birth; you will have a problem when you want to deliver a child because when you do not do sex till you are married you may go through operation because you are small in size so that the baby can come out.”* Combined male and female youths 15-17 years.

Advantages of delaying sexual debut were identified as: no unwanted/early pregnancies, opportunity to complete education uninterrupted, have a secure future, reduced chances of getting STIs, opportunity to enjoy sex at the right time, a girl married as a virgin would be the pride to her family and her future husband and, the ability to plan for the future. Facilitators for delaying sexual debut were identified as; parental teachings, guidance (from parents, teachers, other leaders) and providing for the youth needs, having a good Christian foundation, continuous education and counseling for the youth about disadvantages of early sex and including sex education in the curriculum.

Prevention of STDs especially HIV was the most cited advantage for delaying coirtarche, and there were more advantages for the female more than the male youth.

One religious leader summed up the advantages of delaying sexual debut as follows;

*“…he/she respects the parents and the parents will treat him/ her well as their child who is having self-control and, the second one is that he/she will finish school, … she will not get pregnant and will learn until she finishes school, and the third one is that she will earn respect from the community … the community will rate her in that the daughter of so and so is the best …, the last one her marriage may have a strong foundation because it is a planned one, and there may be a wedding and the person who has married you can also bring many cows because he has married a lady with self-control that is the advantage.*”

### Multiple sexual partners

Generally, participants reported that having more than one partner was common with young people (especially males) in their communities. Peer pressure (competition to have more female sexual partners) for machismo reasons, curiosity to explore the feel of having different sexual partners, and the need to fit into their social groupings are some of the drivers to having multiple sexual partners among young males. Exposure to modernized media including social media, urbanization and access to advanced communication technology (computer, tablets and mobile phones) promote such sexual behaviors in the opinion of some of the older participants (parents, community leaders and religious leaders) as shown below.

“*For most young men and women having sex is more or less like driving a car and everyone wants to have a taste of a Mazda, a Toyota you know next time a Peugeot, a Volkswagen, a Passat what does it feel to drive a Passat (smaller car) and drive a Prado (4-wheel)? … Just like my colleague said they feel you are tougher if you have more sexual partners.*” District Official

While most respondents said there was no difference between male and female youth as far as having multiple sexual partners, some respondents were of the opinion that male youths had a higher tendency to have multiple sexual partners. Those who felt males had a tendency to have multiple sexual partners argued that females were more likely to be faithful and are more serious while males are the ones doing the seduction hence more adventurous.

“*I think when it comes to this relationship mostly men have it higher in that it is being said within the community that there is no woman who is allowed to marry more than one husband but men are allowed to have four or six wives but he is one person.*” Combined Males & Female 18-24.

“*I think males are more aggressive than girls and they also explore more than girls, you may find one male with five girls and he has the ability to lure any girl who is attractive, so out of all these five girls if they agree he will have sex with all of them but a man may attract a lady and it will not be easy for her to approach, so you find that one male in a community can have ten ladies which is unlikely for a woman.*” Community leaders

*“They (male youth) have more partners, you will never find a man having one girl and they know how to play with them, one gets through the backdoor as one enters.”* Combined males and Females 18-24

Apart from traditional gender norms, which prohibits females from having more than one sexual partner, female youth were said to have more chores at home (keeping them busy after school) which deters them from engaging in unfavorable activities while the boys were idle and had more time to mess around.

“*I think males are higher (in number of sexual relations) than females since they have few daily chores as compared to the females and they may find time after school to engage in sexual relationships as compared to ladies who get busy after school.*” Male and female students 17-24 years.

“*The young people think that it is like a weakness when you are a boy. It means that you cannot talk to many ladies. It is a weakness and that is how the society puts it. The young people think that one sexual partner cannot satisfy them so they look for multiple partners*.” Religious leader

Multiple sexual partnerships in males is kind of ‘approved’ by society hence some participants argued that because of this perception, young women become secretive about their sexual activities.

“*If you look into the society then the married men have more ‘mpango wa kando’ (extra marital affairs) as compared to the married women and young women who are not married have multiple partners as compared to the young men who are not married. This is because the one for men is approved by the society but the one for women is associated or tagged with prostitution, so the one for ladies is done secretly. So for those who are not married, they have multiple partners but for the married its men who have multiple partners than women.*” Parents and teachers of youth.

### Perceived advantages and disadvantages of multiple partners

Participants identified what young people perceive as advantages in having multiple sexual partnerships including: prestige (for males); financial gain—receiving support from different partners (for females); an indicator of sexual prowess—ability to seduction and win female sexual partners (for males); reduced stress(options in case of a breakup); having options in case of separation with other sexual partner (permanently or temporarily) and (in polygamous marriage) sharing of responsibilities; and having more children.

“I *think having many partners exposes one to various techniques* [of having sex] *so if you have one partner then you may not have different techniques compared to one who has many.*” Community leader.

Others argued having more than one partner was some kind of ‘security’; that if one relationship ended or the partner moved away then there would always be a fallback plan.

“*Okay it is a way of reducing stress [laughter] in that you may disagree with one then you go to the other one you have a talk, you chat, you do everything so that you can forget what happened with the other one.*” Combined males and females 18-24 years.

*“having more than one sexual partner in a marriage situation means that domestic duties will go on as required and two, you have experience in managing the famil; you get full satisfaction sexually because you are having sex with all partners unless one is sick or has delivered but still the other one will take charge.” C*ommunity Leader

Financial support was a facilitator for some respondents;

“*Me I can see these people, they have many because if I have one boyfriend and he cannot provide all that I need while there is one who can provide then I might double deal.*” Combined males and females 15-17 years

“*Advantage, you can also get a lot of money, let’s say you have even 10 and you tell them you have a problem this one gives you 1000/*= *like that till you get a lot of money.*” Combined males and females 15-17 years

Increased risk of STI, financial burden of supporting many partners (for males), fear of being seen as promiscuous and distrustful were mentioned as factors that dissuade young people from engaging in multiple sexual partnerships as elaborated by one of the young participants.

“*Mmh! Another disadvantage is, if you have many sexual partners you have to also know that he/she is having as many as you have and your chances of getting diseases is high because you have sex with so many people.*” Male and female students 17-24 years’ old.

### Monogamous sexual partnership

Monogamous sexual relationship was said to be facilitated by increased awareness of HIV/AIDS risk and the desire to reduce one’s chance of acquiring STIs especially HIV, financial burden to support multiple sexual partners, religious beliefs and the desire to build trust and maintain one sexual partner.

“*When we flash back, AIDS was not rampant as it is now so to prevent early death you must just have one*.” Community Leader

“*When you have one partner, in case of pregnancy she can know the father of the baby.*” Combined male and female youth 15-17 years’ old

### Perceived advantages and disadvantages of monogamy

Reduced chance of STI, lighter financial burden and being able to only express love emotion to one person and to develop trust and respect in a relationship were cited as benefits of having a monogamous relationship.

*“First it lowers chances of getting HIV since you only have one partner … it also reduces expenses since affairs goes with money” Combined Males & Females 18-24 years.*

“*If you have one you play safe but if you have many then you must get AIDS*.” Females 18-24 years

“A*nother advantage is that the society can talk well about you in that it can be approved by the society even if it reaches time for marriage, then both the parents would approve it very easily compared to a case where they have heard that you have two or three*.” Parents and teachers of youth 18-24.

According to one male youth it is advantageous to have many partners;

On the other hand, lack of “variety” to satiate ones’ sexual libido, relationship insecurity (risk of becoming single and lonely if one separates with a sexual partner), and sense of being trapped in a relationship with one sexual partner were mentioned as some of the disadvantages of monogamy. According to the youth the main disadvantage of having one partner is stress, “*if you have one partner s/he can mistreat or take advantage of you*”.

“A*nother problem is that getting another one will be a problem because any time you go looking for one you will be looking for the gone Adhiambo (female Name) in her and it’s not easy for you to get the one like her and it will take a long time.*” Community Leader

### Intergenerational sexual relationships

Overall, participants were observant of age differences between young men and women in sexual their sexual relationships which could go either way—where the man or woman could be older. While most of the younger participants believed relationships in which the couple are of the same age would not work because women aged faster compared to men, the older participants (community leaders, parents, teachers, etc.) linked intergenerational sex to financial gains.

“*I think in this case mostly men’s ages are a bit higher than ladies in that when you go for a young lady or girl you will choose a young girl because once you go with a girl of your age she will look as if she is your mother*” Combined males and Females 18-24 Years.

They argued that if a couple of the same age got married, either the woman would not respect the man or the man would consider the woman too old (because women mature faster) and therefore there would be constant relationship conflicts. Furthermore, financial considerations accounted for the large age differences observed in some instances with young females and males date older sexual partners for financial as explained by a young female participant below:

“ *… today we are in a small world of dot com. Ladies like us go for sugar daddies so that they can provide and take good care of you instead of going for a youth like you who will just give you stress and all that*” Combined males and females 18-24 Years.

Majority of the participants supported intergenerational sexual relationships where the female is younger. Most of the young participants were of the opinion that young women get involved in sexual relationship with older men because women mature faster and that traditionally, it is the norm that men enter into sexual relationships with women younger than them and that couples of the same age are perceived to be incompatible. Some of the participants went as far as saying younger men do not satisfy the women sexually.

“*I think it depends on what one likes, in our culture a male partner should be older than the female*.” Community Leader

However, some of the participants said that in their communities it was normal and acceptable for young men and women of school/college-going age to have sexual relationship considering that they interact more during their social gatherings (school/college outings, sports, etc.) as described by a participant in the parents’/teacher’s forum:

“*5% are of the same age bracket and 95% are of different age. The 5% is when they are in tertiary college or university they can interact and understand each other because there is some source of income or money that they are given, and when they are outside college the girls are always younger than the males because they get into relationship with older men who can provide for them*” Parents and Teachers of youth 18-24 Years.

Participants were unable to pinpoint the preferred age difference between young men and women saying that in some cases the age difference could be more than 15 or even 30 years (especially where young men/women enter into a relationship for financial benefit). However most of the cited age differences ranged from 1 to 7 years with the smaller age difference in school going youth but increasing as they go to college.

“*You cannot be too definite on the age but it depends. You can even get a person who is 50 years old having an intercourse with 20 years old. It depends with whom this 50 years old person is if the person is rich then age will not be a factor. The age difference can be ten, twenty, and thirty so it depends with who the person is and how sweet he can talk*” Religious leader

Financial gain was cited multiple times as one of the drivers of intergenerational sexual relationships. Besides this, being treated with respect, the belief that women age faster and cultural norm that expects men to be in relationship with woman younger than them, were also mentioned as factors that facilitate intergenerational sexual relationships. The issue of respect was cited in two contexts: that a woman married to an older man will treat him with respect but on the other hand, it was also implied that a mature man knows how to treat and take care of his (younger) woman. The belief that females matured faster was repeatedly mentioned:

“*… sometimes you are age mate to a girl, you find that as time goes the girl grows faster so that when the time reaches for marriage the girl looks a bit older so it will force the man to look for a young girl because the other one* [former partner] *is now older*” Male Youth 15-17 Yrs.

## Discussion

Understanding the perceptions of caregivers, teachers, community leaders and youth themselves about sexual behaviors that put young people at risk of HIV is key in designing prevention interventions that will be acceptable and supported by the youth and the community at large. From the findings above, the youth are believed to be engaging in sex, age of debut is reducing, multiple sexual partnerships are common, and transactional and intergenerational sex are not uncommon.

Early sexual initiation is linked to an increased risk of STIs (including HIV), unintended pregnancies and having multiple sexual partners. Accepting that youth are having sex may be difficult for some parents and policy makers, but it is important to acknowledge the reality of youth sexual activity. Knowledge of the age of sexual debut is key in designing age appropriate intervention programs for these youth who become sexually active at an early age. A study conducted in Moshi, Tanzania among high school students indicated that 21.6% of them had their first sexual encounter before the age of 15 years and over 80% had their sexual debut before they turned 18 years old [31]. The only guaranteed protection against unplanned youth pregnancies and STI/HIV is abstinence, yet delaying coitarche is a challenge to intervention designers and program planners due to peer pressure, a factor that is hard to moderate and, in some cases, economic differentials (poverty and lack of basic needs like sanitary towels) may force young girls to engage in transactional sex. Fear of STIs (mainly HIV) was one of the reasons both male and female youth participants reported delaying sexual debut. For females the main reason to delay sexual debut was fear of pregnancy, which indicates that HIV risk perception is not as high in females.

Past studies have associated peer norms about sexual relationships with sexual behaviors among the youth [16, 18, 46]. Youth are susceptible to peer pressure and are quick to learn certain behavioral patterns from their peers, which affect their attitudes, beliefs, values and choices. Our findings showed that the average age of sexual debut ranged from 12 to 16 years, with peer pressure being cited as the main driver to early sexual debut. In a study that examined potential associations between peer group characteristics, sexual initiation and multiple partnerships among young in Ghana, Bingenheimer et al. [6], established that affiliation with peer groupings and perceived peer norms favoring sex increased the odds of transition to first sex and that having more friends increased the odds of acquiring multiple sexual partners among younger respondents. Similarly, in a qualitative study that sought to understand risks and protective factors against pregnancy amongst sexually-active adolescents in Soweto, South Africa, both male and female group discussants admitted being exposed to a great deal of negative peer pressure surrounding sex and sexual behavior [7]. Since peer pressure is a fundamental factor associated with risky sexual behavior among the youth, there is need to strategize effective interventions that aim at reducing sexual behavior among this sub-population and target them as a group rather than individually.

In this study, the older participants (community leaders, parents, teachers, and religious leaders) believed self-control as a result of good parental guidance can deter early sexual debut. This belief echoes several studies which have shown that positive relationships with such trusted adults have a positive influence on youth sexual behaviors such as delaying sexual debut. Demeke and Sandy [12] in a study exploring factors associated with risky sexual behaviors among youth in Ethiopia identified weak parental control, low level of parental education as negative influencers while attachment to religious institutions as positive influential factors. According to Yakubu and Salisu (2018), some of the factors contributing to adolescent pregnancy in Africa include lack of parental counseling and guidance, and parental neglect, among others. Older respondents also believed more youth were more sexually active compared to the proportion reported by the youth.

A study by Chimah et al. [10] on sexual behavioral pattern of high school students found that girls, at 56.2%, were almost 4-times more likely to have had a forced sexual debut compared to boys, at 14.8%. In a cross-sectional study designed to determine the prevalence of early sexual debut and associated risk factors among high school students in Nigera, of the girls who had initiated sex, almost half (47.5%) confessed to having willingly engaged in early sex while about one-quarter (23.7%) said they did it unwillingly [14]. Religion, parental guidance, role models, having future aspirations and high self-esteem have been identified as protective against early sexual debut [29]. This underscores the fact that youth need social support in terms of supervision and guidance to develop skills for overcoming the pressure of early sexual debut. Parent–child communication about sexual and reproductive health, especially at puberty, is also important in modeling young people’s beliefs, thoughts, and values regarding their sexual health [33]. Research has also shown that staying in school, educational achievements and aspirations protects youth from risky sexual behaviors such as early sexual debut, unprotected sex and having multiple sexual partners [29, 38, 58, 60]. That keeping girls in school reduces both HIV risks and new infections is further supported by UNAIDS in their report which revealed that greater gains were achieved in reducing new HIV infections among adolescent girls and young women in countries with higher school completion [53].

Having multiple sexual partners is one of the well-known risk factors for HIV infection, and is associated with early sexual debut [47]. Environmental factors like prevailing cultural and gender norms influence how youth develop their sexuality hence gender ideologies supporting separate roles for males and females will have an impact on sexuality. From this study, the young men (compared to the women) were far more likely to have multiple sexual partners as a source of pride and gives them perceived higher status among their peers. This concurs with a secondary data analysis by [17] where they found that, compared to their female counterparts, male adolescents were two times more likely to report multiple sexual partners. The findings were from a secondary analysis using data from the Adolescents Module of the cross-sectional household survey on Maternal, Newborn and Child Health. In the community under study it was implied that males having multiple sexual partners was normal (but not for females). This places the female partners in disadvantaged position as they have little or no control over the implications of their partners’ lack of faithfulness. It has been suggested that early sexual debut increases young peoples’ risk for HIV and other STIs [30, 50] given that people who start sexual activity early are more likely to engage in high-risky sexual behavior such as having multiple sexual partners. In a study to determine the association between age at sexual debut and multiple sexual partnership and the mediating role of the knowledge of STIs in the relationship among the youth in Nigeria, Alawode et al. [1] established that adolescents who first had sex at 15 + years were significantly less likely to have multiple sexual partners compared to those who had early sexual debut aged below 15 years. Given the negative outcomes associated with multiple sexual partnerships, understanding why young people engage in multiple sexual partners is an important consideration in the efforts aimed at changing such behavior and tackling its outcome.

Intergenerational transactional sex in the community under study is driven mainly by widespread poverty and limited employment opportunities. In this study, both young men and women (but mostly women) confessed to engaging in sexual relationships with much older partners for financial gain. Zamudio-Haas et al. [59] in their qualitative study with young people aged 15–19 years in informal settlement communities in Kisumu, Kenya, found that poverty was the main driver of intergenerational sex. The term transactional sex generally refers to exchange of sexual services in return for compensation which could be money, clothing or even status [28]. In most cases, people who engage in transactional sex do not consider themselves as sex workers. Studies have suggested that transactional sex could be a risk factor for HIV infection in females [24, 40, 55]. Furthermore, Wamoyi et al. [55] in their systematic review and meta-analysis assessing the relationship between transactional sex and HIV among men and women in sub-Saharan Africa found that women who practice transactional are nearly 2 times more likely to be infected with HIV. However, association between transactional sex and HIV infection among men was inconclusive. In Kenya, the relatively well-off older partner is referred to as a "sponsor." Factors leading to this risky sexual behavior in young men and women therefore need to be understood and addressed. Casual relationships with older rich men offer the young girls resources to increase their attractiveness to potential suitors and accord them a higher social status among their peers. Disadvantaged women from low socio-economic status have less negotiation power and therefore likely to be coerced into engaging in transactional sex [55, 59].

Female youth are at a higher risk of HIV infections and to influence youth sexual behavior, HIV prevention strategies need to address gender vulnerabilities, as well as promoting a protective environment for women. Inequality and powerlessness prevent women from protecting themselves hence the need to have in place supporting socio-structural programs such as promoting girl child education, alongside the behavioral interventions. On the other hand, more advantaged women may use their status to influence relations with relatively younger men as suggested by some participants, further underscoring the case for poverty alleviation and structural interventions. Cultural gender norms perpetuating the relative powerlessness and therefore vulnerability of the female youth need to be addressed in intervention programs promoting sexual behavior change for youth. Interventions need to acknowledge the perceived benefits of intergenerational transactional sexual relationships. Interventions should target the structural constraints young women face and support them to access resources, empower them to reject advances and not depend on men for their financial needs.

Delaying sexual debut, reducing the number of sexual partners and avoiding age mixing are all ways of preventing HIV infections in youth. However, when it comes to stopping the pandemic, no single method offers the magic bullet and there is consensus that application of combination prevention methods is a viable solution to the HIV pandemic. Such approaches need to be youth friendly and informed by and relevant to the local cultures and norms.

## Conclusion

The study revealed that the youth commonly engaged in pre-marital sex and regularly had multiple sexual partners (especially males). Age mixing/intergenerational transactional sex was also widespread among youth of both genders. Youth in high HIV burden settings are engaging in sexual behaviors that put them at risk of STIs/HIV as well as unintended pregnancies. These behaviors are facilitated by social-economic, cultural and religious factors, which must be considered when designing HIV prevention interventions targeting this population.

Barriers to educating the youth about HIV and reproductive health in general are multidimensional and include individual, cultural and religious among others. The unique concerns of the youth must be taken into consideration for them to benefit from HIV prevention programs. Peer pressure was a recurring individual barrier to sexual behavior change hence priority interventions targeting this group should consider outreach through peer educators. Supporting the youth to acquire skills and knowledge that will help them deal with peer pressure, male sexual prowess attitudes, and poverty, in tandem with improving communication between children and parents are key to reducing the three sexual behavioral risks discussed in this paper. These strategies have the potential to significantly boost HIV prevention efforts for youth by creating gender specific, age appropriate interventions. HIV risk perception also needs to be raised. Female youth were more worried about pregnancy than HIV infection if they engaged in sexual intercourse. There is plenty of room for improvement in HIV prevention for youth in Kenya, especially in Homabay County.

Bearing in mind that not all youth may be willing or able to abstain, HIV prevention and reproductive health programs for youth should still emphasize abstinence and debunk myths that pressure youths towards early sexual debut while transmitting messages about reducing the number of sexual partners and practicing safe sex.

## Data Availability

The datasets generated and analyzed during the study are available in the Impact Research and Development Organization (IRDO) repository.
